# A Double-Blind Randomized Controlled Trial Comparing Intrathecal 0.5% Isobaric Levobupivacaine With Fentanyl to 0.5% Isobaric Ropivacaine With Fentanyl for Elective Cesarean Section

**DOI:** 10.7759/cureus.76846

**Published:** 2025-01-03

**Authors:** Disha Juneja, Kalpana Verma, Sapna Singh, Mangilal Deganwa

**Affiliations:** 1 Anesthesiology, Mahatma Gandhi Medical College and Research Institute, Jaipur, IND; 2 Anesthesia and Critical Care, Mahatma Gandhi Medical College and Research Institute, Jaipur, IND

**Keywords:** isobaric levobupivacaine, isobaric ropivacaine, motor block, sensory block, spinal anesthesia

## Abstract

Background

This study was planned to compare the onset and duration of action and the levels of sensory and motor blocks of 0.5% isobaric levobupivacaine with fentanyl to 0.5% isobaric ropivacaine with fentanyl addition in subarachnoid block in elective cesarean cases.

Materials and methods

This hospital-based randomized interventional controlled study was conducted in a tertiary care facility in Jaipur. Sixty women who planned for elective lower-segment cesarean section, with more than 37 weeks of gestational period during the study period, were included in our study. These women were randomly divided into two study groups, using sealed opaque envelopes, and double blinding was ensured. In group L, women received 0.5% isobaric levobupivacaine with fentanyl, and in group R, 0.5% isobaric ropivacaine with fentanyl was given.

Results

The mean time for sensory and motor block onset was significantly lower in group L than in group R (p<0.05). The mean time for two-segment regression for sensory block was significantly higher in group L than in group R (p<0.05). The mean duration of total motor blockade (B0) was significantly higher in group L compared to group R. The differences in the mean duration of total motor blockade were statistically significant (p<0.05). The mean change in mean arterial pressure (MAP) in group L was significantly higher than in group R (p<0.05).

Conclusion

This study provides valuable insights into the comparative effects of 0.5% isobaric levobupivacaine with fentanyl and 0.5% isobaric ropivacaine with fentanyl for spinal anesthesia in cesarean sections. Both anesthetic agents were well-tolerated, with no severe complications or side effects observed in either group. While levobupivacaine offers an early onset of action, ropivacaine demonstrates a more favorable hemodynamic profile with an early regression of motor block. Ropivacaine may be a suitable alternative to levobupivacaine for spinal anesthesia in cesarean sections, particularly in patients with unstable hemodynamics. Further research is necessary to investigate the long-term effects of these agents and to optimize their use in different patient populations.

## Introduction

Spinal and epidural anesthesia are commonly used for cesarean deliveries as they offer several advantages. These techniques reduce the risk of maternal mortality and morbidity by maintaining the patient's consciousness and intact airway [1]. This minimizes the need for airway manipulation, thereby lowering the risk of aspiration, postoperative complications, deep vein thrombosis, pulmonary embolism, and pneumonia [2]. Common local anesthetics used for spinal anesthesia include lignocaine, bupivacaine, levobupivacaine, and ropivacaine.

Levobupivacaine, the pure S-enantiomer of bupivacaine, exhibits a safer pharmacological profile due to its faster rate of protein binding and lower affinity for cardiac sodium channels, resulting in fewer deleterious cardiac and neurotoxic effects. Moreover, compared to racemic bupivacaine, levobupivacaine leads to an earlier motor recovery [3-6]. Ropivacaine, an amino-amide local anesthetic, shares structural similarities with mepivacaine and bupivacaine, but it is 30%-40% less potent than bupivacaine [7]. This drug was developed with the aim of reducing the risk of cardiovascular toxicity and enhancing the relative sensory and motor block profiles compared to previous local anesthetics. Since ropivacaine has a shorter duration of action and is well-tolerated following intrathecal administration, it may serve as a suitable substitute for lidocaine in ambulatory surgery [8].

Comparing isobaric ropivacaine to bupivacaine at the same doses revealed that ropivacaine was almost as effective but caused a shorter period of sensory and motor block [9]. Following the subarachnoid injection of 0.5% ropivacaine, the parameters of the sensory block are comparable to those of 0.5% bupivacaine, while ropivacaine provides a less effective motor block compared to bupivacaine [10,11].

Fentanyl has been utilized in conjunction with local anesthetics to improve analgesia while minimizing the effects on motor and sympathetic blocks of spinal anesthesia. This has led to a reduction in the incidence of hypotension, as well as earlier recovery and mobilization. Additionally, it has allowed for a decrease in the amount of local anesthetic needed [12,13].

This study was planned with the aim of comparing the onset and duration of action, as well as the levels of sensory and motor blocks, and adverse events of 0.5% isobaric levobupivacaine with fentanyl to 0.5% isobaric ropivacaine with fentanyl addition in subarachnoid block in elective cesarean cases.

## Materials and methods

This hospital-based randomized controlled study was conducted in the Department of Anesthesia of Mahatma Gandhi Medical College and Hospital, Jaipur, after approval from the Institutional Ethical Committee (approval number: 1624) and registration under the clinical trial registration number CTRI/2024/02/063381.

Study design

This is a double-blind randomized controlled study.

Study type

This is a hospital-based, interventional study.

Study period

Data collection for the study was started as per the aims and objectives after getting approvals from the Institutional Ethical Committee and Central Trial Registry of India for the period of three months from March 2024 to May 2024.

Study population

A total of 60 women were included in the present study with elective lower-segment cesarean section of more than 37 weeks of gestational period. The population size was calculated using a finite population and Z score (Z score=1.96 at 0.95 confidence interval). Women who had contraindications to spinal anesthesia; those who had received medications other than perinatal vitamins, calcium, protein, and iron preparations; those with systemic disorders; pregnant mothers with fetal abnormality, placenta previa, and abruption placenta; and patients who refused to be a part of the experiment were excluded from the study.

Sixty enrolled women were randomly divided into two study groups, using sealed opaque envelopes, and double blinding was ensured: group L, 10 mg 0.5% isobaric levobupivacaine (2.0 mL)+25 mcg fentanyl (0.5 mL); group R, 10 mg 0.5% isobaric ropivacaine (2.0 mL)+25 mcg fentanyl (0.5 mL).

Procedure

A standard American Society of Anesthesiologists (ASA) monitoring, wide-bore intravenous access was established using an 18-gauge (G) intravenous cannula, and a simultaneous infusion of 15 mL/kg of Ringer's lactate solution was administered intravenously. Antiaspiration prophylaxis was established. The subarachnoid block was carried out with meticulous aseptic measures, while the patient was positioned in a seated posture. Using a midline approach, a 25 G Quincke's needle was inserted into the subarachnoid space at either the L3-L4 or L4-L5 interspinous space. The needle was advanced until the resistance was no longer felt, and the cerebrospinal fluid (CSF) freely flowed into the hub of the spinal needle, confirming successful placement. Next, a volume of 2.5 mL of the study medication solution was administered intrathecally, meaning it was injected into the spinal canal. The injection was done slowly over a period of 10 seconds, with the bevel of the needle facing toward the head. The time of medication administration was recorded, and all subsequent observations were referenced to this time as "zero" minutes. Without delay, the patient was positioned on their back and given oxygen at a rate of 5 L/minute using a face mask. Ringer's lactate solution was employed to maintain fluid balance and replenish lost blood.

Sensory characteristics were evaluated and documented both during and after the surgery. The duration for the block to reach the T8 segment is considered as the initiation of the sensory block. The duration of time required to reach the maximum degree of the block is recorded as the interval between the intrathecal injection and the attainment of the highest sensory level. The time required for a two-segment regression of the sensory level is measured as the duration of the sensory block. The motor block is evaluated using the modified Bromage scale, which ranges from 0 to 3. The initiation of motor block is classified as modified Bromage grade 3. Patients were also monitored for the occurrence of any adverse effects such as nausea, vomiting, hypotension, bradycardia, and difficulty breathing for a period of 24 hours after the procedure.

Statistical analysis

Data thus collected were entered in a Microsoft Excel sheet (Microsoft Corp., Redmond, WA). Continuous/quantitative data were summarized in the form of mean and standard deviation. The significance of the difference between the two means was analyzed using a paired t-test. Discrete/qualitative data were summarized in the form of proportion. The significance of the difference in proportion was analyzed using the chi-square test. The level of significance was set at 5% for all statistical analyses.

## Results

Age distribution

The chi-square is equal to 4.510 with two degrees of freedom; the P value is equal to 0.105.

In our study, the mean age of cases in group L was 27.60±3.71 years, while in group R, it was 26.67±3.14 years (Table 1). Both study groups were comparable in terms of age distribution (p>0.05).

**Table 1:** Age-wise distribution of cases

Age groups (years)	Group L	Group R
21-25	10 (33.3)	11 (36.7)
26-30	12 (40)	17 (56.7)
31-35	8 (26.7)	2 (6.7)
Grand total	30 (100)	30 (100)

Sensory and motor block characteristics

The mean time for the onset of sensory block was 1.29±0.12 minutes in group L and 1.51±0.22 minutes in group R. The mean time for the onset of motor block was 12.82±1.59 minutes in group L and 15.83±2.45 minutes in group R. The mean time for two-segment regression for the sensory block was 163.33±10.61 minutes in group L and 86.83±9.42 minutes in group R. The mean duration of total motor blockade (B0) was 139.7±8.88 minutes and 69.73±7.02 minutes in groups L and R, respectively. The mean time to first rescue analgesia was 132.63±10.57 minutes in group L and 129±12.21 minutes in group R. The differences in the mean onset times for sensory and motor blocks, the time for two-segment regression for sensory block, and the duration of total motor blockade were statistically significant (p<0.05). However, the difference in the mean time to first rescue analgesia was not statistically significant (p>0.05) (Table 2).

**Table 2:** Average time for block characteristics of the participants The P value was calculated using a paired t-test

Variables	Group L	Group R	P value
Time of the onset of sensory block (in minutes)	1.29±0.12	1.51±0.22	<0.001
Time of the onset of motor block (Bromage scale B3)	12.82±1.59	15.83±2.45	<0.001
Two-segment regression for sensory block (in minutes)	163.33±10.61	86.83±9.42	<0.001
Total duration of motor blockage (B0) (in minutes)	139.7±8.88	69.73±7.02	<0.001
Time to first rescue analgesia (in minutes)	132.63±10.57	129±12.21	0.223

Incidence of itching

In group L, nine of the cases experienced itching, compared to five in group R. The difference in the proportion of cases with itching between the two groups was not statistically significant (p>0.05) (Table 3). The chi-square is equal to 0.839 with one degree of freedom; the P value is equal to 0.360.

**Table 3:** Incidence of Itching in both study groups

	Group L	Group R
Itching	9 (30)	5 (16.7)
No itching	21 (70)	25 (83.3)
Grand total	30 (100)	30 (100)

The preoperative mean arterial pressure (MAP) of patients in both groups was at par with the mean arterial pressure (MAP) of 87.04±5.85 of group L and 90.40±6.54 of group R, while the MAP after the spinal anesthesia of group L was 76.91±4.58 and group R 84.23±6.54. The postoperative MAP in group L was 92.28±7.67 and in group R 88.78±6.58. The mean change in MAP in group L was 27.14±3.65 compared to 4.55±1.12 in group R. This difference was found to be statistically significant (p<0.01) (Table 4).

**Table 4:** Changes in mean arterial pressure (MAP) in both groups The P value was calculated using a paired t-test SP, systolic pressure; DP, diastolic pressure

Variables	Group L	Group R	P value
Preoperative (SP)	118.25±8.54	122.14±9.58	0.049
Preoperative (DP)	71.44±5.41	74.54±4.56	0.036
MAP (preoperative)	87.04±5.85	90.40±6.54	0.019
After spinal anesthesia (SP)	100.45±7.54	112.45±8.95	0.002
After spinal anesthesia (DP)	65.14±3.56	70.12±4.14	0.034
MAP (after spinal anesthesia)	76.91±4.58	84.23±6.54	0.01
Postoperative (SP)	133.78±11.25	119.24±8.12	0.001
Postoperative (DP)	71.54±5.42	73.56±5.23	0.56
MAP (postoperative)	92.28±7.67	88.78±6.58	0.047
Changes in MAP (postoperative and preoperative)	27.14±3.65	4.55±1.12	0.001

Complications

In the current study, among the participants of group L, around one-third of them have postoperative bradycardia, and 26.7% of them have shivering, while among group R, 13.3% have bradycardia, and 20% have shivering. The difference in the proportion of cases with bradycardia and shivering was statistically insignificant (p>0.05). In group L, five (16.7%) participants have no postoperative complications, and 16 (53.3%) participants in group R have no complications. This difference in proportion among both study groups was found to be statistically significant (p<0.05) (Table 5).

**Table 5:** Proportions of complications among both study groups The P value was calculated using the chi-square test

Complications	Group L	Group R	P value
Bradycardia	11 (36.7)	4 (13.3)	0.074
Shivering	8 (26.7)	6 (20)	0.760
No complication	5 (16.7)	16 (53.3)	0.026

## Discussion

The choice of anesthesia technique for surgery depends on factors such as the type of surgery, its duration, the patient's health, and potential complications. Spinal anesthesia is a popular option, offering a quick onset, suitable surgical anesthesia duration, and rapid recovery with minimal side effects.

While various intrathecal drugs have been explored, their use has been limited due to potential cardiac and neurological side effects, as well as delayed recovery. This study aims to compare the clinical effects of 0.5% isobaric levobupivacaine to 0.5% isobaric ropivacaine, both with the addition of intrathecal fentanyl, in spinal anesthesia for elective cesarean sections. Previous studies have reported that isobaric levobupivacaine and isobaric ropivacaine at 0.5% concentration have been optimized for maximum efficacy [14].

To ensure that differences in drug metabolism and action due to age were not confounding factors, the study groups were matched for age. The study participants were evenly distributed across different age groups in both study groups.

The onset of sensory and motor block was faster with levobupivacaine compared to ropivacaine. The mean time to the onset of sensory block for levobupivacaine was 0.22 minutes faster than ropivacaine. Similarly, the mean time to the onset of motor block was 3.01 minutes faster for levobupivacaine as compared to ropivacaine. The increased lipid solubility and formulation of levobupivacaine likely contributed to its faster onset of action. Similar results were obtained by Selvin et al. [15], Samar et al. [16], ​​​​​and Athar et al. [17].

Motor block in spinal anesthesia is the loss of motor function that occurs when local anesthetics block nerve impulses in the motor nerve fibers. In the present study, the mean duration of motor block by levobupivacaine was significantly high as compared to ropivacaine. Similar findings were reported by Selvin et al. [15] and Athar et al. [17].

Time to regression in spinal anesthesia is the time interval between the injection of an intrathecal agent and the time when the sensory block regresses. In the current investigation, the mean time of two-segment regression was longer in group L than in group R, and this difference was statistically significant. Kuusniemi et al. also reported a higher regression time for levobupivacaine compared to ropivacaine, which aligns with the findings of our study [18].

The mean arterial pressure (MAP) is the average blood pressure during a single heartbeat. It provides a crucial measure of how effectively blood is circulating through the body's tissues. In this study, the mean change in MAP was significantly lower in the ropivacaine group compared to the levobupivacaine group. This suggests that ropivacaine offers greater hemodynamic stability. This difference can be attributed to ropivacaine's lower lipid solubility compared to levobupivacaine, resulting in a less toxic effect on the cardiovascular system and heart [19]. Thus, this drug can be used in patients with a history of deep vein thrombosis or preexisting cardiac comorbidities.

The patients in the present study were evaluated and closely observed for complications due to spinal anesthesia. It was observed that no severe complications occurred in either group L or group R, indicating that both drugs are safe to use. Similar findings were reported by Joseph and Philip [20]. Furthermore, no severe side effects of the drugs were observed, and no failed spinal anesthesia was reported.

This investigation highlights the use of isobaric ropivacaine with fentanyl as an anesthetic, which can be useful for mothers with unstable hemodynamics and to reduce changes in mean arterial pressure intra- and postoperatively. Further long-term experiments with a higher number of patients are required to fully understand the mechanism of both drugs. Different concentrations of both drugs can be tested in order to properly evaluate their working and side effects.

The study's single-center design may restrict the generalizability of its findings to broader populations or settings, as the results might not fully reflect diverse geographic or demographic variations. Additionally, the relatively small sample size could limit the study's statistical power and introduce a larger margin of error, potentially impacting the reliability of the conclusions. By focusing on neonatal assessment, the study does not address longer-term outcomes or effects, creating gaps in understanding the broader implications. Moreover, the restricted scope of the assessment may have overlooked other critical variables or factors that could influence the findings.

## Conclusions

This study provides valuable insights into the comparative effects of 0.5% isobaric levobupivacaine with fentanyl and 0.5% isobaric ropivacaine with fentanyl for spinal anesthesia in cesarean sections. Both anesthetic agents were well-tolerated, with no severe complications or side effects observed in either group. While levobupivacaine offers an early onset of action, ropivacaine demonstrates a more favorable hemodynamic profile with an early regression of motor block. Ropivacaine may be a suitable alternative to levobupivacaine for spinal anesthesia in cesarean sections, particularly in patients with unstable hemodynamics. Further research is necessary to investigate the long-term effects of these agents and to optimize their use in different patient populations.
